# Use of lorazepam for analgosedation during mechanical ventilation in pediatric intensive care

**DOI:** 10.3389/fmed.2025.1600671

**Published:** 2026-01-23

**Authors:** Paul Healy, Marco Marano, Marcello Montibeller, Bianca Maria Goffredo, Giuseppe Pontrelli, Oscar Della Pasqua

**Affiliations:** 1 Clinical Pharmacology & Therapeutics Group, University College London, London, United Kingdom; 2 Ospedale Pediatrico Bambino Gesù (OPBG), Rome, Italy

**Keywords:** lorazepam, dose rationale, analgosedation, pharmacokinetic modeling, pediatric extrapolation, repurposing

## Abstract

**Introduction:**

Lorazepam has been used off-label for analgosedation in pediatric intensive care units (PICU) as an alternative to midazolam. While its intermediate duration of action makes it suitable for continuous sedation, there is limited evidence to guide dosing in children. This study illustrates how pharmacokinetic modeling and extrapolation principles can be used to (1) identify regimens that maintain the desired analgosedation levels and (2) optimize the design of a prospective protocol in children requiring mechanical ventilation.

**Methods:**

Pharmacokinetic data and COMFORT-B scores from a preliminary pilot study in six mechanically-ventilated pediatric patients (aged 0.8–4.8 years) were available for the purpose of the current investigation. A previously published population pharmacokinetic model was used to characterize the disposition of lorazepam, accounting for developmental growth and metabolic maturation in children. Parameter distributions were used as priors. Clinical trial simulations (CTS) were subsequently performed in a virtual cohort of 100 children (aged 1.0–12 years) to explore optimized dosing regimens, combining intermittent bolus dosing and continuous infusions over a 72-h period. A target concentration of 500 ng/ml was selected considering the available clinical data and literature evidence on the analgosedative effects and safety profile of lorazepam. Simulation scenarios also explored sample size and sampling time requirements for a prospective clinical trial.

**Results:**

The pharmacokinetic model adequately described the concentration vs. time profiles, despite appreciable interindividual variability. Population estimates for clearance and volume of distribution were 0.23 L/h/kg and 2.3 L/kg, respectively. Simulation results showed that intermittent bolus dosing every 4 h, followed by continuous infusion allowed for lorazepam steady state concentrations to fluctuate around 500 ng/ml. An initial dose of 0.2 mg/kg given as bolus every 4 h over the first 24 h, followed by a similar regimen with 0.1 mg/kg over the subsequent 24 h and continuous infusion of 0.03 mg/kg/h until the end mechanical ventilation was identified as the recommended regimen to be evaluated in a prospective clinical trial.

**Conclusion:**

Our study underscores the importance of model-based approaches to identify suitable dosing regimens to be used in children when limited pharmacokinetic and pharmacodynamic data are available. The proposed dosing regimen balances efficacy and safety data, thereby offering the foundation for the repurposing of lorazepam as an alternative, second line option for analgosedation of mechanically ventilated subjects in a pediatric intensive care unit setting.

## Introduction

Sedation and analgesia are necessary components of the treatment of critically ill patients admitted to a pediatric intensive care unit (PICU), and their purpose is to provide a continuous level of patient comfort. This is essential during intubation and mechanical ventilation, but controlling pain and agitation and reducing discomfort are equally important. Current guidelines emphasize the need for the routine monitoring of pain, agitation, withdrawal, and delirium using validated tools and enhanced use of protocolised analgesia and sedation, i.e., with prioritization of pain relief before adding sedatives. The specific criteria for adding benzodiazepines are to manage anxiety or facilitate tolerance of procedures, after adequate analgesia has been achieved (1, 2).

Whilst different, personalized approaches may be required in different settings, there are limited data on the optimal degree of sedation for more complex cases, such as critically ill patients or those undergoing extracorporeal membrane oxygenation (3, 4). Multiple sedative options are available to the pediatric critical care provider, including alpha_2_-agonists, benzodiazepines, propofol, and ketamine. Yet, fentanyl and midazolam are reported as the preferred combination for analgosedation and reduction of pain and discomfort in mechanically ventilated patients. (5–7). In fact, the extensive use of benzodiazepines in PICU is due to their excellent efficacy profile and low toxicity, despite the potential for delirium and iatrogenic withdrawal syndrome when patients are not adequately monitored (1).

Even though analgosedation with alpha_2_-agonists has been associated with a reduction in opioid requirements, as compared to midazolam in mechanically ventilated PICU patients (1), lorazepam may offer a suitable alternative, as it has a longer half-life and more predictable pharmacokinetics without the concern of active metabolites (8, 9). Lorazepam has an intermediate duration of activity following intravenous administration as a continuous infusion or intermittent bolus. Its metabolism is primarily determined by conjugation (i.e., glucuronidation) (10). Consequently, lorazepam biotransformation does not result in active metabolites, and as such it has low potential for drug-drug interactions. It has been shown that it is significantly easier to induce and maintain a predefined level of sedation with lorazepam than with midazolam (11). In addition, these characteristics allow better prediction of time to wakening after infusion discontinuation.

For many years, lorazepam has been used intravenously as an antiepileptic medication for status epilepticus in children at doses of 0.1 mg/kg up to a maximum of 4 mg, with concentrations in plasma reaching approximately 100 ng/ml and effective range between 30 and 50 ng/ml for 6–12 h (12). In fact, only recently, the European Medicines Agency (EMA) approved the inclusion of control of status epilepticus in adults, adolescents and children from 1 month old to the summary of product characteristics (13). On the other hand, the use of lorazepam remains contraindicated in children under 12 years of age due to the lack of safety data in children. Such concerns arise from the potential risk of toxicity in young children exposed to the combination of benzyl alcohol, propylene glycol and polyethylene glycol, which are used as excipients in the formulation. Consequently, for the treatment of status epilepticus, the EMA advises not to exceed the 4 mg dose within 24 h in children (14).

Lorazepam has also been authorized for the sedation of adults to induce anterograde amnesia, at a dosage up to 0.06 mg/kg to be administered as a bolus in 15–20 min, with a maximum total dosage of 4 mg. Despite the anticipated benefits of lorazepam in analgosedation in patients admitted to a PICU, its use remains off-label, with limited data on its pharmacokinetics and pharmacodynamics in this population. In children, doses ranging from 0.05 to 0.15 mg/kg/h have been reported in the literature (15, 16). However, these regimens have not taken into account the nonlinearity in age- and weight-related changes in drug disposition. In most cases, safety considerations have prevailed over a more structured assessment of the benefit-risk ratio. Consequently, focus has been given to the exposure to propylene glycol. Previous studies suggest that propylene glycol serum concentrations correlate positively with the dose, duration or rate of infusion of lorazepam (17–19). This, together with a substantial lack of information of the effects of mechanical ventilation on the disposition characteristics of lorazepam, makes it essential to evaluate the pharmacokinetics of lorazepam in pediatric patients in a PICU setting. Pharmacokinetic data can provide a robust basis for the dose selection and identify the most appropriate regimen (infusion rate) for safe and effective analgosedation.

Based on a retrospective review of clinical cases in patients, during which lorazepam has been used off-label (20–22), the indicative threshold for obtaining effective analgosedation appears to occur at higher concentrations (i.e., 100–500 ng/ml) than those required for the treatment of status epilepticus. In these clinical cases, the use of intermittent boluses allowed for faster increase in plasma lorazepam concentrations, and consequently resulted in a faster onset of sedation. In addition, it is worth noting that this range is below the levels that have been associated with high probability of delirium in adult patients in an intensive care setting (i.e., >500 ng/ml) (23, 24). Further details on the pharmacological and clinical basis for the selected target range are presented the subsequent sections.

Taking into account the role of developmental growth and maturation processes on the pharmacokinetics of lorazepam, here we apply modeling and simulation concepts to analyse data from small cohort of patients treated off-label with lorazepam as basis for the selection of suitable dosing regimens for analgosedation in patients admitted to a PICU. The aim of the present investigation was therefore to characterize the pharmacokinetics of lorazepam and provide recommendations for the dose and design of a prospective, controlled study in which lorazepam is used in lieu of midazolam in mechanically ventilated pediatric patients in a PICU setting.

Given the lengthy, time-consuming process required for the approval of its use in status epilepticus in children (25), we also hope to illustrate how modeling, simulation and extrapolation principles can be applied to facilitate drug repurposing and label extension for pediatric diseases (26). The proposed approach has been used in other therapeutic areas (27–29), and allows for the evaluation of clinically relevant scenarios, supporting data integration and evidence generation in a more effective manner than what is implemented empirically in traditional clinical trials.

## Methods and materials

### Clinical data

Pharmacokinetic data from a pilot cohort (*n* = 6) was available for the purpose of this investigation (Table 1). In this pilot study, the pharmacokinetics, safety and analgosedative effect of different doses of lorazepam (0.1–0.2 mg/kg), administered either as bolus (20 min) or continuous infusion (up to 72 h) were evaluated. Patients were subject to mechanical ventilation throughout the treatment period. Sparse blood samples (an average of six per patient) were collected between 10 min and 84 h after the start of the administration of lorazepam. The study was approved by the Ethics Committee of the Bambino Gesù Pediatric Hospital, Rome, Italy and conducted in accordance with the guidelines of the Declaration of Helsinki. Informed consent was provided by each participating infant's parents or legal guardian.

**Table 1:** Baseline characteristics of the study cohort (*n* = 6).

Median age in years (range)	1.1 (0.88–4.86)
Median weight in kilograms (range)	11.0 (5.7–15.0)
Sex (male, female)	*M* = 3, *F* = 3

### Bioanalytical assay

Lorazepam plasma levels were measured in the Laboratory of Metabolic Diseases and Drug Biology at Bambino Gesù Pediatric Hospital. Plasma was recovered by centrifuging EDTA-tubes containing whole blood at 3,500× g for 5 min and stored at −80 °C until processing. Liquid chromatography and mass spectrometry analysis were performed by using an UHPLC Agilent 1290 Infinity II coupled to a 6470 Mass Spectrometry system (Agilent Technologies, Deutschland GmbH, Waldbronn, Germany) equipped with an ESI-JET-STREAM source operating in the positive ion (ESI+) mode adapted from (30). A MassHunter Workstation v. 10.1 (Agilent Technologies) was used for controlling this equipment and analyzing the data. Calibrators, quality controls (QCs), and plasma samples were analyzed using a validated LC-MS/MS kit (ClinMass^®^ TDM Kit System) provided by RECIPE Chemicals + Instruments GmbH (Dessauerstraße 3, 80992 München, Germany). The assay calibration curve was linear for lorazepam and ranged from 2.50 to 2,500 ng/ml. The limit of detection (LOD) and lower limit of quantification (LLOQ) defined by this kit were 0.83 and 2.50 ng/ml, respectively. Plasma samples were prepared for the analysis following the manufacturer's instructions. Samples with a lorazepam concentration above the higher calibration point were further diluted with drug-free plasma obtained from healthy donors and re-analyzed once again. Each batch of patients' analyses included both low and high QCs at fixed concentrations of 60 and 200 ng/ml, respectively. The commercial kit included plasma lyophilized calibrators and QCs and was validated according to the ICH M10 guideline on bioanalytical method validation and study sample analysis. The intra- and inter-assays precision, defined as mean % coefficient of variation (CV), was determined from 10 independent runs for each QC level over a period of four months. In particular, for the intra-assay precision the %CV was 2.80 and 5.40 for low and high QCs, respectively. Similarly, the inter-assay %CV was 5.30 for low and 6.20 for high QCs. The recovery rate for all 35 benzodiazepines included in this kit lies between 87.7 and 101 %. These data agreed with the acceptance criteria established by the EMA guideline on bioanalytical method validation and study sample analysis.

### Pharmacokinetic modeling, simulation and dose optimisation

**Figure 1:**
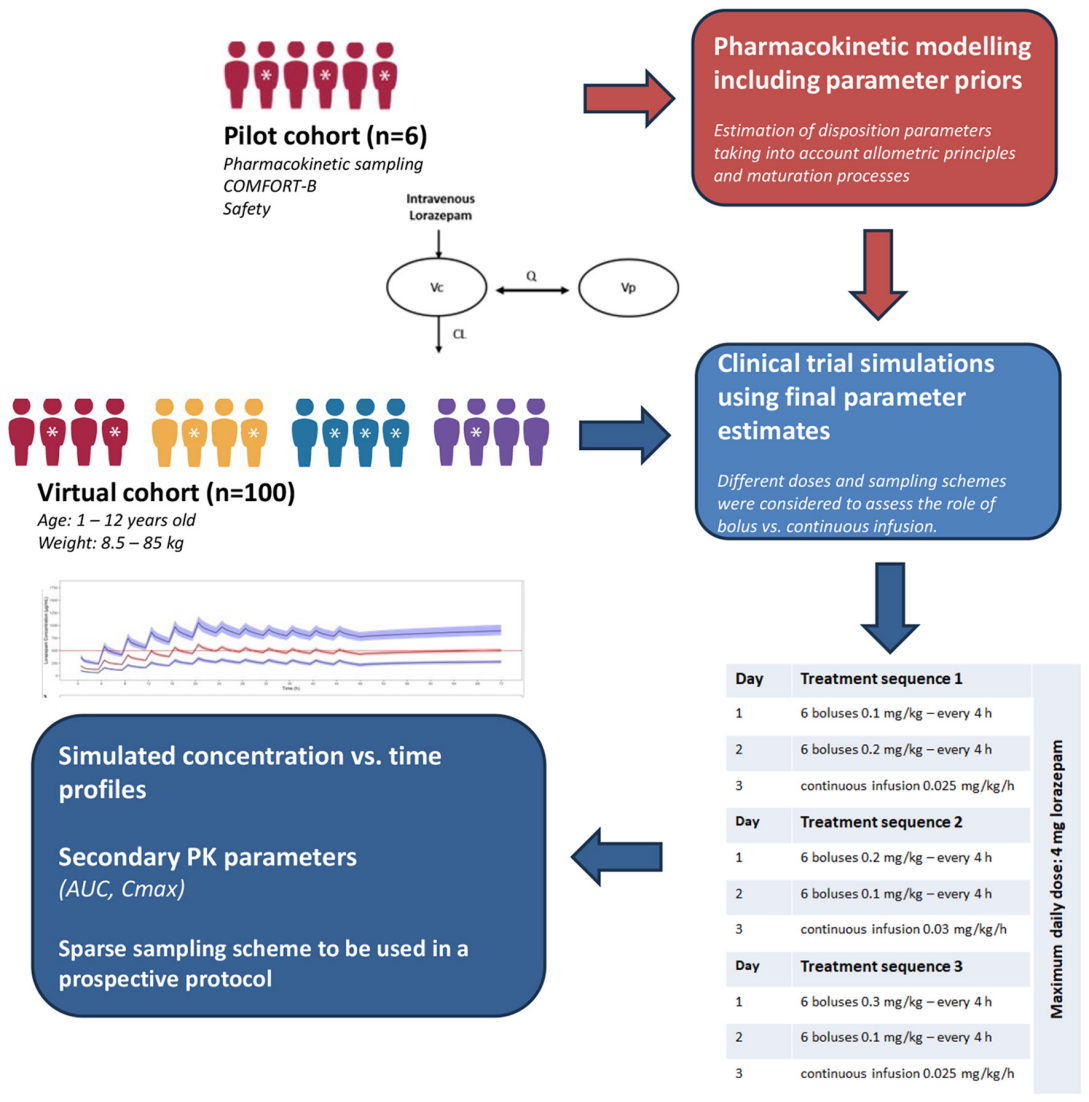
Workflow of the steps taken in our analysis from modeling of the clinical data to the implementation of clinical trial simulations aimed at assessing optimised dosing regimens of lorazepam.

To characterize the disposition of lorazepam and evaluate optimized dosing regimens in pediatric patients, we followed a structured workflow, incorporating data analysis, model development, and clinical trial simulations. This overall process is illustrated in Figure 1.

### Population pharmacokinetics

**Figure 2:**
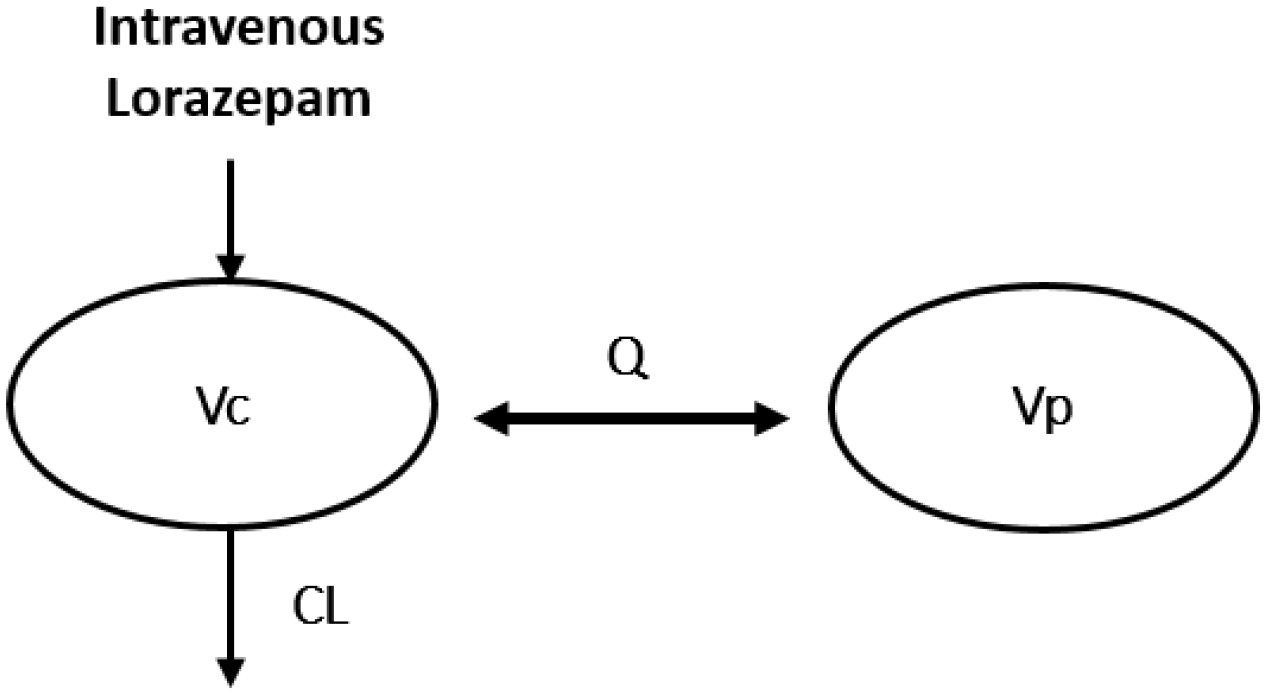
Compartmental model used for the analysis of lorazepam (31). CL, clearance; Q, intercompartmental clearance; Vc and Vp, central and peripheral volumes of distribution.

The pharmacokinetic data were analyzed using a non-linear mixed effects model previously published by Gonzalez et al. (31) in which the disposition properties of lorazepam were characterized in children by a two-compartment model (Figure 2). As a sparse blood sampling scheme was used in the pilot study, the model was adapted to incorporate priors to support parameter estimation (32). Therefore, model structure and covariates (i.e., body weight and post-natal age) were implemented as reported. The effect of body weight on clearance (CL), intercompartmental clearance (Q) and volume of distribution (V1) was described by an allometric function, whereas an exponential function was used to assess the effect of post-natal age on clearance. The following relationships characterized the typical values for the central compartment volume (V1), CL, peripheral compartment volume (V2), and intercompartmental clearance (Q): V1 (L) = 0.879^*^WT; CL (L/h) = 0.115^*^(Age/4.7) 0.133^*^WT^0.75^; V2 (L) = 0.542^*^V1; Q (L/h) = 1.45^*^WT^0.75^. The first order conditional estimation with interaction (FOCE-I) option and ADVAN 3 TRANS4 was used with the $PRIOR function. During model evaluation, inspection of the goodness of fit plots showed a few observations with large conditional residual errors that met the criteria for the outliers. We have therefore decided to remove each one at a time, in a stepwise manner based on the magnitude of the conditional residual errors. In total, these observations represented up to 17% of the collected blood samples. No separate covariate analysis was attempted as the data were too sparse to identify additional covariate effects.

Model performance was assessed by goodness-of-fit plots of population (PRED) and individual (IPRED) predictions vs. observed concentrations (DV) and conditional weighted residuals (CWRES) vs. time and PRED. Individual visual predictive checks (VPCs) were implemented by simulating concentration vs. time profiles for each individual (500 replicates), and plotting the resulting 90% prediction interval, along with the observed concentrations.

### Virtual population

As the scope of this investigation was to identify suitable regimens to be evaluated in a small prospective clinical trial, attention was also paid to the study design optimisation, including sampling times and sample size (33, 34). To this end, a virtual cohort including 100 pediatric patients was created using individual-level demographic data with variables including age, sex and weight from the National Health and Nutrition Examination Survey (NHANES) (35) and CALIPER (36) databases. Our cohort was created by sampling randomly (*n* = 100) from a lager pooled patient dataset (*n* = 1,000) and used for the implementation of clinical trial simulations, with body weight uniformly distributed across the different treatment arms (Table 2). Clinical and practical aspects were considered for the selection of relevant simulation scenarios (Figure 1). The assessment criteria were based on a similar approach to that described previously for dose selection and protocol optimisation in different acute and chronic pediatric indications (37, 38). In addition, the virtual cohort was also used to assess model performance by replicating the published concentration vs. time plots by Gonzalez et al. (31), which were digitized and overlaid to the predicted profiles.

**Table 2:** Relevant patient baseline demographic characteristics of the virtual cohort of patients (*n* = 100) used in the simulation scenarios.

Age (years)	8 (1.2–12)
Weight (kg)	29.1 (8.9–87.4)

Figures are shown as median and range.

### Simulation assumptions

As the current evaluation is part of a broader investigation aimed at identifying the benefit-risk balance of lorazepam for analgosedation, a common set of assumptions has been used taking into account the presence of propylene glycol (14, 39). Eight key assumptions were required for the evaluation and interpretation of the results, namely:

1. Sedation protocols for mechanically ventilated PICU patients have been based on reported scores for clinical scales, such as the State Behavioral Scale (SBS), the COMFORT behavior scale (COMFORT-B scale), the Ramsay sedation scale, the Richmond Agitation Sedation Scale (RASS) and Nurse Interpretation of Sedation Score (NISS) (40, 41). Here we assume a nonlinear relationship between plasma concentration and degree or level of sedation, even though estimates of drug potency (IC_50_) and maximum effect are limited to a post-operative setting in adult patients.
2. Given that the exposure-analgosedation (PKPD) relationship has not been characterized for the proposed indication, a therapeutic target range has been derived based on steady state concentrations, ensuring that the predicted median lorazepam concentrations remain above that required for the management of status epilepticus and below values known to be associated with delirium (12, 24).
3. Dose proportionality (i.e., pharmacokinetic linearity) was assumed within the proposed dose range, even if distribution delays may occur due to haemodynamic factors associated with mechanical ventilation. In addition, it can be anticipated that its pharmacokinetics is relatively insensitive to changes in hepatic blood flow (11). The same assumptions were applied to describe dose-related changes in exposure to propylene glycol.
4. Given that lorazepam is metabolized via a non-oxidative route, and converted into a pharmacologically inactive metabolite (3-O-glucoronide), the original model parameterisation based on total body weight and age was deemed adequate to describe maturation and size-dependent changes in drug disposition (42).
5. Interindividual variability in disposition properties was based on previously reported data in children. In addition, it is assumed that lorazepam shows relatively low variability due to its limited hepatic extraction and route of elimination. The potential effect of interindividual differences in conjugating hepatic enzyme systems and the corresponding changes due to critical illness was deemed to be minor (11).
6. The threshold of 500 ng/ml as a target for effective analgosedation was selected based on clinical case reviews, interindividual variability in response to benzodiazepines and literature supporting higher plasma concentrations than those needed for seizure control (typically 30–50 ng/ml) (12). Adult ICU studies have shown that plasma levels exceeding 500 ng/ml may be associated with increased delirium risk (24), while concentrations between 100–500 ng/ml are thought to reflect effective sedation with an acceptable safety profile (11, 24). This range encompasses the mean IC_50_ values for moderate sedation (i.e., 152 ng/ml) in mechanically ventilated adult ICU patients (41). Thus, maintaining exposure below or near this boundary aims to ensure effective sedation while mitigating toxicity risk.
7. Differences of up to 15% in median secondary pharmacokinetic parameter estimates (AUC, Cmax, and Cmin) were not deemed clinically relevant. Such a variation allows for the effect of model uncertainty whilst taking into account the impact of circadian changes in nociception (43).
8. Treatment interruptions were considered to be unlikely within the selected dose range. Similarly, scenarios in which supplementary analgosedation was required due to sub-optimal sedation, were not simulated.

### Software and applications

All data handling, statistical and graphical summaries were conducted in R (v. 3.6.1), using the graphical interface R-studio (v1.2.5019) (44). Model development was implemented using NONMEM v 7.4. and the Perl-speaks-NONMEM (PsN v.4.9.0) library of modules (45, 46).

## Results

**Figure 3:**
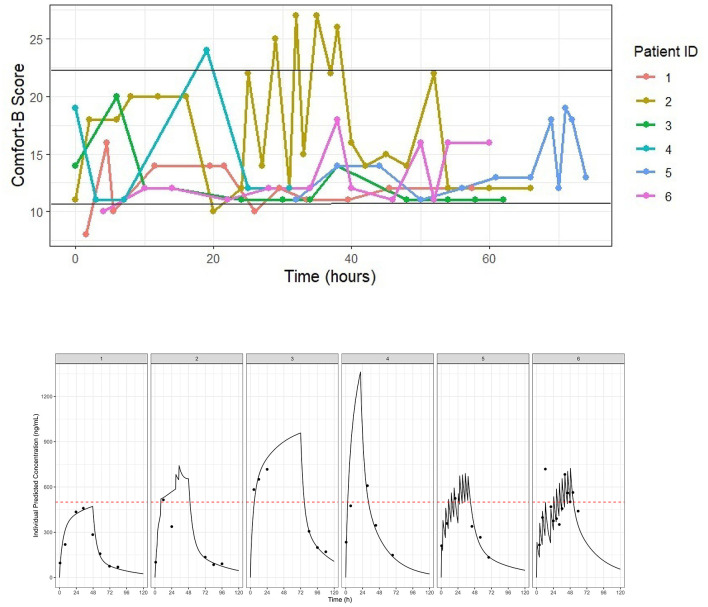
**(Upper panel)** Individual Comfort-B scores over the course of analgosedation with lorazepam. Each line represents one subject. Horizontal black lines depict the interval associated with optimum sedation (i.e., Comfort-B scores between 11 and 22) (52, 53). Despite high interindividual variability most patients reached and maintained adequate analgosedation throughout the study. **(Lower panel)** Individual concentration vs. time profiles of lorazepam in six mechanically ventilated patients undergoing analgosedation. The solid black lines represent the individual predicted profiles, whilst the dots indicate the observed plasma concentrations. The dashed red line marks the target median concentration (500 ng/ml), which is proposed to achieve adequate analgosedation in pediatric patients.

**Figure 4:**
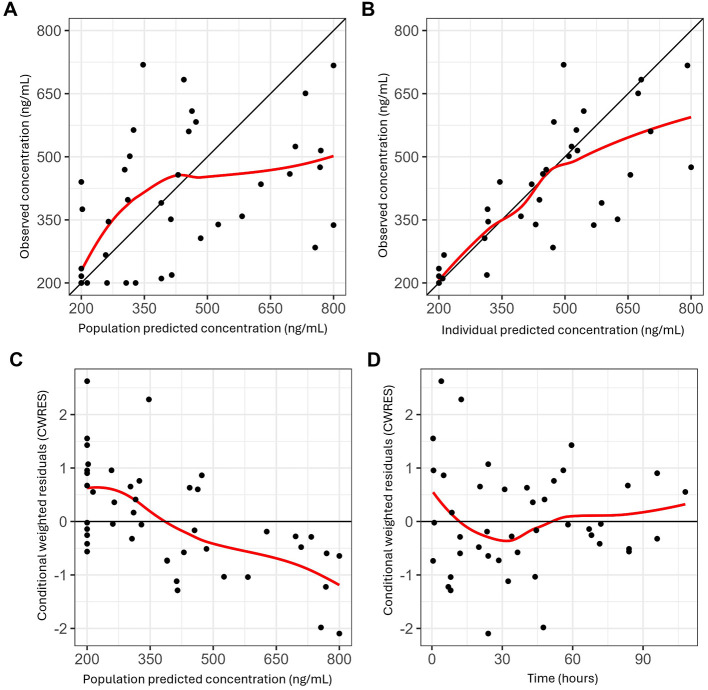
Panels **(A)** and **(B)** show, respectively, the observed versus population and individual predicted lorazepam concentrations, with black dots representing the observed data. Panels **(C)** and **(D)** depict the conditional weighted residuals (CWRES) plotted against population-predicted concentration and time. The red line is a loess smoothed trend, while the diagonal line represents the line of unity. Even though no specific explanation was identified for the shift in the trend line at high concentrations, this could be associated with haemodynamic or vascular changes caused by potentially critical illness.

**Figure 5:**
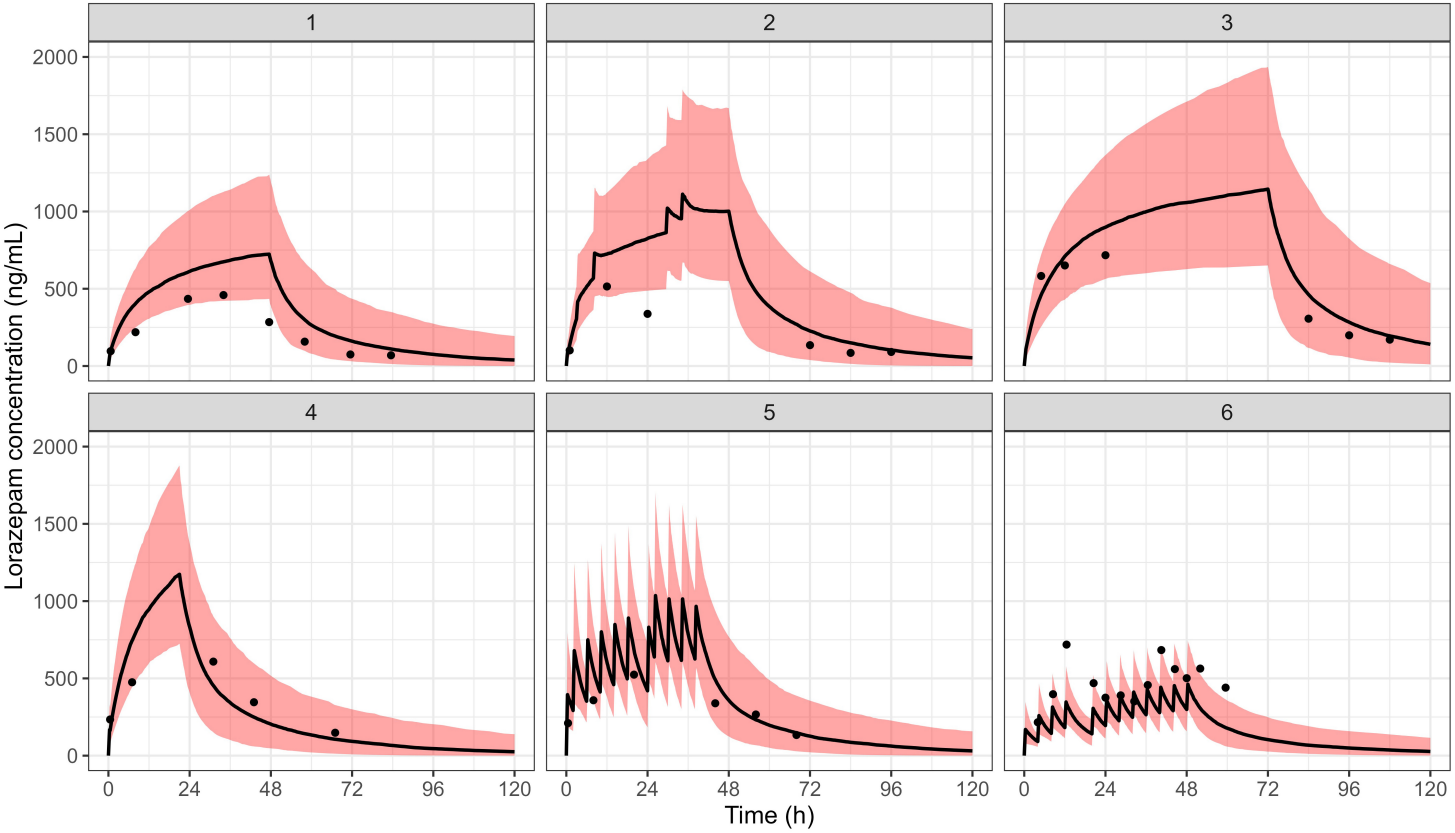
Individual visual predictive checks (VPCs). The solid red line represents the median predicted plasma concentration vs. time profile of lorazepam, whilst the black dots are the observed data. Shaded areas represent 90% prediction intervals.

Despite high inter-individual variability in the pharmacokinetics and pharmacodynamics of lorazepam, adequate analgosedation was achieved throughout the pilot study. Individual Comfort-B scores remained within the interval associated with optimum sedation (i.e., Comfort-B scores between 11 and 22) most of the time during which patients were mechanically-ventilated (Figure 3, upper panel). The pharmacokinetics of lorazepam in this group was adequately described by a two-compartment model with first order elimination. As anticipated, prior parameter distributions were required to overcome convergence issues due to the small sample size. The individual predicted lorazepam concentration vs. time profiles for each of the six patients are shown in Figure 3 (lower panel). The typical population value for clearance (CL) was 0.232 L/h/kg, with a central volume of distribution (Vc) of 1.150 L. Inter-individual variability (IIV) on clearance and volume was moderate, with shrinkage below 20% for these parameters. Residual variability was described by a proportional error model. Goodness-of-fit diagnostics showed acceptable model performance, providing a satisfactory description of the data. As illustrated in Figure 4, observed and predicted concentrations showed a reasonable correlation, but a trend toward underprediction of lorazepam concentrations greater than 500 ng/ml remained in the conditional weighted residuals (CWRES). This trend, however, was not observed in the individual visual predictive checks (VPCs). As shown in Figure 5, the median predicted plasma concentrations were found to be consistent with the observed data. The majority of the observed concentrations fell within the 90% prediction interval. The final population pharmacokinetic parameters are summarized in Table 3.

**Table 3:** Overview of the secondary pharmacokinetic parameters of lorazepam, including the mean, median, standard deviation, and percentiles (5^th^ and 95^th^) for the maximum concentration (Cmax) and area under the curve (AUC) across the different simulation scenarios.

**Variable**	**Mean**	**Median**	**Standard deviation**	**Percentile [5^th^]**	**Percentile [95^th^]**
**Sequence 1** ***Bolus (0–24 h)***					
Cmax	327.1	310.4	116.1	168.4	541.2
AUC	4501.4	4238.8	1692.4	2240.5	7642.5
**Sequence 1** ***Bolus (24–48 h)***					
Cmax	766.6	729.3	257.8	417.1	1242.9
AUC	12992.7	12311.6	4530.4	6916.4	21383.8
**Sequence 1** ***Infusion (48–72 h)***					
Cmax	617.5	584.1	219.6	324.7	1026
AUC	13460.8	12725.2	5025.8	6728	22802.3
**Sequence 2** ***Bolus (0–24 h)***					
Cmax	654.1	620.8	232.2	336.8	1082.4
AUC	9002.8	8477.5	3384.8	4480.9	15285.1
**Sequence 2** ***Bolus (24–48 h)***					
Cmax	616.9	585	208.9	337.4	1004
AUC	12096.1	11448.6	4327.8	6343.4	20135
**Sequence 2** ***Infusion (48–72 h)***					
Cmax	535.8	504.6	201	271.9	914.1
AUC	12106.5	11416.3	4540.7	6096.1	20603.8
**Sequence 3** ***Bolus (0–24 h)***					
Cmax	981.2	931.2	348.3	505.2	1623.6
AUC	13504.2	12716.3	5077.2	6721.4	22927.5
**Sequence 3** ***Bolus (24–48 h)***					
Cmax	852.2	807.6	298.3	451.5	1404.8
AUC	15829.3	14982.3	5816.7	8021.4	26587.4
**Sequence 3** ***Infusion (48–72 h)***					
Cmax	563.8	531.4	230.4	253.3	993.7
AUC	12990.7	12194.5	5351.1	5874.5	23020.4

**Figure 6:**
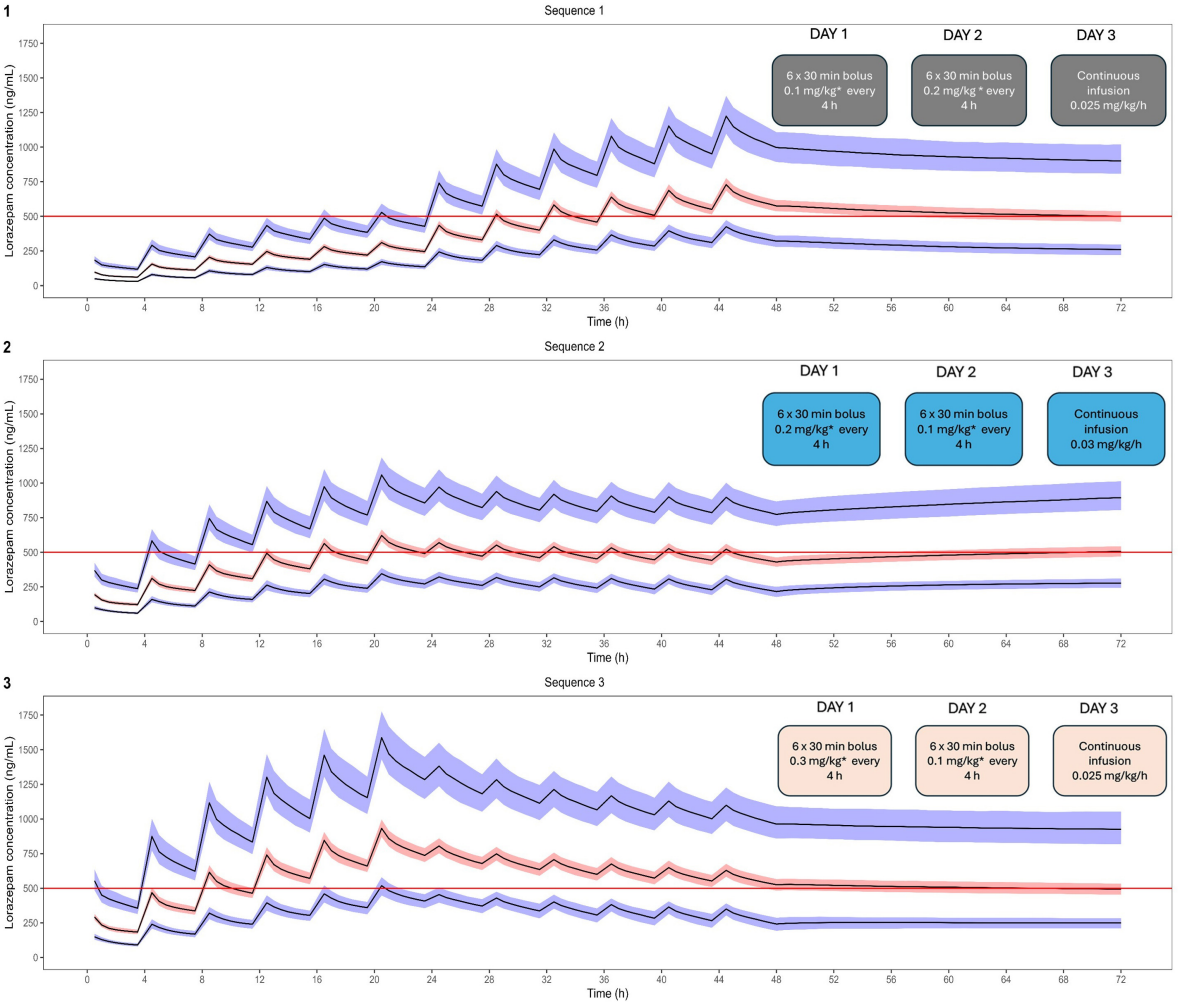
Simulated concentration vs. time profiles after administration of different doses and dosing regimens of lorazepam, delivered as bolus and continuous infusion, as defined in the simulation scenarios for treatment sequences 1, 2 and 3, which are outlined in the panel inlets. The solid lines depict the 5^th^, 50^th^, and 95^th^ percentiles and corresponding 90% prediction intervals obtained from 500 simulations. Horizontal red line indicates the median target concentration.

**Figure 7:**
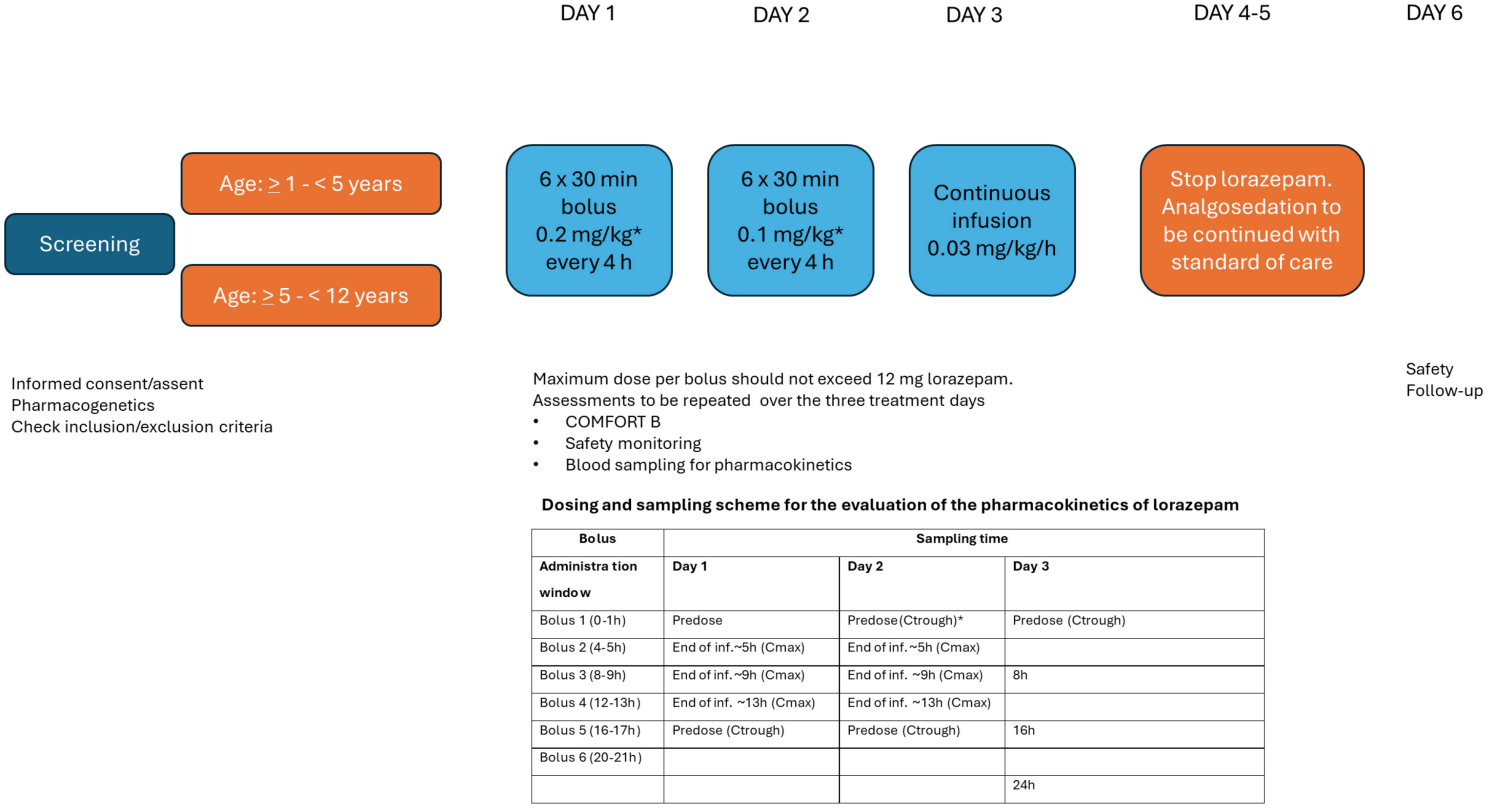
Proposed protocol design for the evaluation of the analgosedative effects, safety profile and pharmacokinetics of lorazepam in pediatric patients from 1 to 12 years old undergoing mechanical ventilation. A sample size of 12 evaluable subjects with 6 subjects per age group should provide estimates with sufficient precision to determine the suitability of the proposed dosing regimen for analgosedation. *Bolus administration at the planned intervals takes place as long as there is no evidence of excessive analgosedation, as assessed by the COMFORT-B scores. Intervals can be adjusted to prevent over or under-sedation.

The final model parameters were then used to explore the impact of different dosing regimens on the systemic exposure to lorazepam in a virtual cohort of 100 children (aged 1.2–12 years). Figure 6 depicts the changes in the concentration vs. time profiles following bolus dosing and various rates of infusion. Three dosing sequences were evaluated: intermittent bolus dosing every 4 h followed by continuous infusion for 48–72 h. The simulated scenarios revealed that intermittent bolus dosing in combination with continuous infusion allowed for faster attainment of target concentrations compared to continuous infusion alone. Table 3 summarizes the secondary pharmacokinetic parameters associated with each regimen, including the mean, median, and percentiles for maximum concentration (Cmax) and area under the curve (AUC). Sequence 2 (0.2 mg/kg bolus every 4 h followed by a continuous infusion of 0.03 mg/kg/h) appeared to result in the most stable profile, with median target concentrations around 500 ng/ml throughout the 72-h period, which are likely to minimize the risk of under or overexposure. Based on the current results, an initial draft protocol design was proposed for the implementation of a prospective clinical trial for the evaluation of analgosedation in a group of 12 pediatric patients undergoing mechanical ventilation (Figure 7).

## Discussion

Conducting clinical pharmacology (PKPD) studies in children, especially in critically ill patients, is fraught with ethical and practical challenges, which limit the possibility of empirical evidence generation. This is compounded by limitations of current sedation practice, which include serious adverse drug events, prolonged mechanical ventilation time, and longer stay at the intensive care unit. By contrast, this study presents the application of modeling, simulation and extrapolation principles as a tool for the optimisation of the dosing regimens of lorazepam in the analgosedation of pediatric patients undergoing mechanical ventilation. In addition to providing an opportunity for the integration of available pharmacokinetic and pharmacodynamic data, our analysis shows how clinical trial simulations can be used to support the design of study protocols involving patients in a pediatric intensive care unit. Moreover, it illustrates how innovative computational and experimental methodologies can be combined, thereby overcoming common challenges in pediatric drug repurposing (27, 33).

Here, we have focused on the use of prior distributions to characterize drug disposition based on sparse data from a small cohort of pediatric patients (47). More specifically, model structure identification, including covariate factors would not be accurately defined without such an approach, given the role of multiple concurrent factors on drug disposition properties (48–50). Of interest in our analysis was also to assess whether mechanical ventilation and other PICU procedures would result in significant alteration of the pharmacokinetics of lorazepam. A previous study in pediatric patients with and without status epilepticus reported a median clearance and volume of distribution values of 1.08 (range 0.3–7.75) ml/min/kg and 1.37 (range 0.49–3.40) L/kg, respectively (12). Even though a wider age range was enrolled into the study, these figures are in agreement with our results, which showed a median value of 3.86 ml/min/kg for clearance and 2.3 L/kg for the volume of distribution. These results suggest minor or no impact of mechanical ventilation and consequently changes in hemodynamics on drug disposition parameters, which reflects the pharmacokinetic features of lorazepam, i.e., a low extraction ratio drug. On the other hand, it worth mentioning that median clearance estimates normalized by body weight in adults is significantly lower 1.03 L/min/kg, indicating the effect of increased metabolism in children.

Even though clinical guidelines emphasize the importance of prioritizing pain management with opiates and alpha agonists for analagosedation in a PICU setting, considering benzodiazepines as second line, the use of benzodiazepines still needs to be carefully evaluated given the heterogeneity in patient response (1–3). Previous reports show that analgosedation results in improved patient outcomes compared to standard sedative-hypnotic regimens (1, 51). It is conceivable that the gap in understanding of the concentration-effect relationship of lorazepam, and potential risk of toxicity associated with the presence of propylene glycols have prevented further evaluation of its role in analgosedation. Clearly, there is an opportunity to titrate lorazepam to the desired level of sedation, as assessed by the COMFORT scale (52–55). In fact, different attempts have been made to gather conclusive evidence of the performance of lorazepam in a PICU setting (21, 22). Unfortunately, the results from a Phase II/III clinical study aimed at the evaluation of the sedative effect of lorazepam in critically ill children (*n* = 179), including pharmacokinetic and pharmacodynamic endpoints, has never been made publicly available (NCT00109395) (56).

Nevertheless, we have attempted to correlate our results with the findings by de Wildt and colleagues, who reported adequate analgosedation following the administration of midazolam at a median infusion rate of 0.09 mg/kg/h (range 0.05–0.4 mg/kg/h) (57). Our recommendation for the use of continuous infusion at a rate of 0.03 mg/kg/h seems to correspond to the known higher binding (affinity) of lorazepam to the benzodiazepine receptors, i.e., reflecting the fact that lorazepam shows approximately two- to three-fold higher potency than midazolam. In addition, it is worth mentioning that the high variability in analgosedation observed in the current study seems to reflect the lack of a clear relationship between drug concentrations in plasma and pharmacodynamic effects in critical care patients. The same phenomenon was reported for midazolam (57).

Despite its higher potency and longer elimination half-life than midazolam, which may be clinically useful in mechanically ventilated patients, lorazepam does not appear to be frequently used in PICUs across Europe, as highlighted in a recent survey on pain and sedation management and monitoring of the Pharmacology Section and the Nurse Science Section of the European Society of Pediatric and Neonatal Intensive Care (6), In this survey, 71% of the units refer to the use of protocols for analgosedation management based on a combination of an opioid and a benzodiazepine, with fentanyl and midazolam being the preferred drugs. On the other hand, lorazepam has been used as a weaning medication in pediatric patients exposed to opioids or intravenous midazolam (15, 58). Such a practice suggests that there have been limited safety concerns at the selected dose range.

Based on the aforementioned, the implementation of clinical trial simulations underscores, beyond doubt, the utility of leveraging quantitative clinical pharmacology methodologies to address gaps in current understanding and overcome some of the practical constraints in pediatric research, especially when considering vulnerable populations, such as critically ill patients. Incorporating prior knowledge and optimizing trial design, including virtual cohorts and different intervention scenarios, substantially improves the quality and informative value of evidence generated from small-scale studies. Whilst pediatricians and intensive care professionals may not be familiar with the use of *in silico* clinical trials as a design optimisation tool, our approach aligns with the evolving regulatory strategic framework and reflects key principles outlined in guidelines for pediatric drug development. More specifically, it illustrates how innovative methodologies can be applied to repurposing or label extension of approved medicines (59, 60). It also provides an example of how modeling, simulation and extrapolation can be used for the development of medicinal products for the pediatric population (61, 62).

Lastly, we recognize some limitations in our study, which extend beyond the small sample size. First, we have had to exclude a few samples (outliers) to adequately characterize the pharmacokinetics of lorazepam in this population. We could not exclude the effect of potential discrepancies between nominal and actual catheter dead volume, timing and duration of bolus infusions, etc., which may occur due to the clinical setting within a pediatric intensive care unit. These factors add to the known contribution of age-dependent changes in hepatic glucuronidation capacity, body composition, organ function, and differences in haemodynamic status due to potentially critical illness. Consequently, we understand that prospective data collection will be required to further assess the performance of the model in very young children (i.e., <2 years old), for whom post-natal age may not adequately describe the variability in ontogeny-related processes. We also acknowledge that parameter estimates may deviate from those obtained in the simulation-re-estimation procedures that were used to optimize the sampling scheme and select dosing regimens for the prospective study. However, it can be anticipated that a more stringent monitoring can be implemented in a randomized study setting. In addition, we have not performed an integrated analysis of pharmacokinetic and pharmacodynamic data. Rather, we relied on inferences from available data on analgosedation with midazolam, taking into account differences in the relative potency between the two moieties. Obviously, such inferences do not incorporate variability in analgosedation due to neurological and neurodevelopmental factors, co-morbidities as well as concomitant medications (61). By implementing an optimized protocol design, in which pharmacokinetic, efficacy and safety data are collected, as recommended in Figure 7, it may be possible to assess the clinical implication of the different regimens and clarify whether a more flexible titration schedule is required.

## Conclusions

Our study underlines the importance of model-based approaches in optimizing dosing regimens when limited pharmacokinetic and pharmacodynamic data are available. The proposed dosing regimens attempt to balance efficacy and safety, thereby offering the foundation for the repurposing of lorazepam as a potential second line option for analgosedation in a pediatric intensive care unit setting. We realize, however, that satisfactory analgosedation may require titration and tapering steps, to ensure appropriate management of interindividual differences in pharmacodynamics. Undoubtedly, the pharmacokinetics and safety profile of propylene glycol should also be further evaluated.

## Data Availability

The raw data supporting the conclusions of this article will be made available by the authors, upon reasonable request.
